# Clinical Categorization Algorithm (CLICAL) and Machine Learning Approach (SRF-CLICAL) to Predict Clinical Benefit to Immunotherapy in Metastatic Melanoma Patients: Real-World Evidence from the Istituto Nazionale Tumori IRCCS Fondazione Pascale, Napoli, Italy

**DOI:** 10.3390/cancers13164164

**Published:** 2021-08-19

**Authors:** Gabriele Madonna, Giuseppe V. Masucci, Mariaelena Capone, Domenico Mallardo, Antonio Maria Grimaldi, Ester Simeone, Vito Vanella, Lucia Festino, Marco Palla, Luigi Scarpato, Marilena Tuffanelli, Grazia D’angelo, Lisa Villabona, Isabelle Krakowski, Hanna Eriksson, Felipe Simao, Rolf Lewensohn, Paolo Antonio Ascierto

**Affiliations:** 1 Melanoma, Cancer Immunotherapy and Development Therapeutics Unit, Istituto Nazionale Tumori IRCCS Fondazione G. Pascale, 80131 Napoli, Italy; g.madonna@istitutotumori.na.it (G.M.); me.capone@istitutotumori.na.it (M.C.); d.mallardo@istitutotumori.na.it (D.M.); a.grimaldi@istitutotumori.na.it (A.M.G.); e.simeone@istitutotumori.na.it (E.S.); v.vanella@istitutotumori.na.it (V.V.); l.festino@istitutotumori.na.it (L.F.); m.palla@istitutotumori.na.it (M.P.); l.scarpato@istitutotumori.na.it (L.S.); m.tuffanelli@istitutotumori.na.it (M.T.); grazia.dangelo@istitutotumori.na.it (G.D.); 2 Theme Cancer, Karolinska University Hospital, 171 76 Stockholm, Sweden; giuseppe.masucci@ki.se (G.V.M.); lisa.villabona@ki.se (L.V.); hanna.eriksson@sll.se (H.E.); rolf.lewensohn@ki.se (R.L.); 3 Department of Oncology and Pathology, Karolinska Institutet, 171 64 Stockholm, Sweden; isabelle.krakowski@ki.se; 4 Theme Inflammation, Karolinska University Hospital, 171 76 Stockholm, Sweden; 5 Genevia Technologies OY, 33100 Tampere, Finland; felipe.simao@geneviatechnologies.com

**Keywords:** ipilimumab, nivolumab, pembrolizumab, melanoma, checkpoint inhibitors, BRAF/MEK inhibitors, survival random forest model

## Abstract

**Simple Summary:**

Immune checkpoint inhibitors have improved the prognosis for patients with advanced melanoma. Despite the recent success of immunotherapy, many patients still do not benefit from these treatments, and their real-life application may yield different outcomes compared to the advantage presented in clinical trials. There is therefore a need to select patients who can really benefit from these treatments. We have focused our study on a real-life retrospective analysis of metastatic melanoma patients treated with immunotherapy at a single institution—the Istituto Nazionale Tumori IRCCS Fondazione “G. Pascale” of Napoli, Italy. With the help of AI and machine learning we validated an algorithm based on clinical variables of patients—namely, the Clinical Categorization Algorithm (CLICAL)—that defines five predictable cohorts of benefit to immunotherapy with 95% accuracy. It can be a useful tool for the stratification of metastatic melanoma patients who may or may not improve from immunotherapy treatment.

**Abstract:**

The real-life application of immune checkpoint inhibitors (ICIs) may yield different outcomes compared to the benefit presented in clinical trials. For this reason, there is a need to define the group of patients that may benefit from treatment. We retrospectively investigated 578 metastatic melanoma patients treated with ICIs at the Istituto Nazionale Tumori IRCCS Fondazione “G. Pascale” of Napoli, Italy (INT-NA). To compare patients’ clinical variables (i.e., age, lactate dehydrogenase (LDH), neutrophil–lymphocyte ratio (NLR), eosinophil, BRAF status, previous treatment) and their predictive and prognostic power in a comprehensive, non-hierarchical manner, a clinical categorization algorithm (CLICAL) was defined and validated by the application of a machine learning algorithm—survival random forest (SRF-CLICAL). The comprehensive analysis of the clinical parameters by log risk-based algorithms resulted in predictive signatures that could identify groups of patients with great benefit or not, regardless of the ICI received. From a real-life retrospective analysis of metastatic melanoma patients, we generated and validated an algorithm based on machine learning that could assist with the clinical decision of whether or not to apply ICI therapy by defining five signatures of predictability with 95% accuracy.

## 1. Introduction

In the past 10 years, we have seen the evolution of melanoma treatment attributed to the development of novel immunotherapy agents that target specific immune regulatory checkpoints, which have completely changed the perspective for metastatic melanoma patients by increasing survival rates and improving quality of life [1,2,3,4,5,6]. In this context, two immune inhibitory molecules involved in immunosuppressive response have been actively studied: cytotoxic T-lymphocyte-associated antigen-4 (CTLA-4), which helps to extinguish the immune activator signal, and programmed cell death protein 1 (PD-1), which negatively regulates T-cell activation and inhibition of effector function [7]. Based on evidence from prior studies, the idea was born that specific antibodies suppressing inhibition of the immune system in the cancer microenvironment could prevent the inactivation of an effector antitumor immune response [8]. Ipilimumab—a monoclonal antibody (IgG1) directed against CTLA-4—was the first immunotherapy for metastatic melanoma to have shown a benefit to overall survival (OS) in approximately 20% of patients in a randomized phase III trial [9]. Nivolumab and pembrolizumab—monoclonal antibodies directed against PD-1—are other immunomodulating agents able to reactivate innate antitumor immunity, eliciting objective responses in a substantial percentage of patients with melanoma [10,11,12,13]. However, only a portion of patients will benefit from immunotherapies and, although many studies have been carried out to identify potential predictive and/or prognostic biomarkers useful to identify patients who respond to therapies [14,15,16], no universally recognized biomarkers are available to date. There is a lack of prognostic biomarkers, and this is one of the main limitations affecting the use of these immunomodulating antibodies. There are described prognostic clinical variables, but there are no comprehensive ways to keep them under a common denominator related to the benefit of interactive treatment. Some examples have been previously described [17,18,19]. In addition, most efficacy data related to the use of immunomodulating antibodies are derived from randomized trials; their real-life application might give different outcomes compared to the results from clinical trials, as the inclusion and exclusion criteria might be selective and give overoptimistic survival rates. Here, we present real-world data related to 578 metastatic melanoma patients treated at the INT-NA with the immune checkpoint inhibitors (ICIs) ipilimumab, nivolumab, or pembrolizumab as monotherapies to investigate whether patients’ baseline clinical characteristics could predict their response to treatment.

## 2. Materials and Methods

### 2.1. Study Population

**Figure 1:**
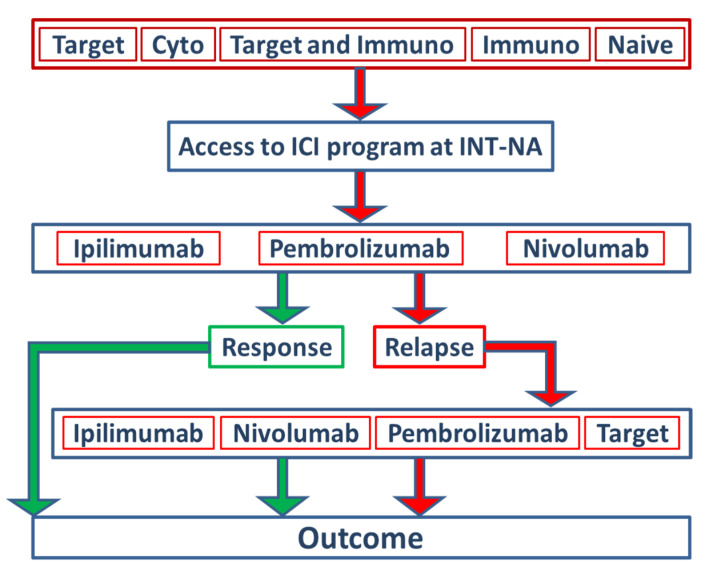
Study design and stratification of patients within the ICI program at INT-NA. In total, 578 stage IV melanoma patients treated with ipilimumab (51%), pembrolizumab (23%) or nivolumab (26%) as monotherapies at the INT-NA were included in the present study. Based on the type of treatment received before inclusion in the ICI program at the INT-NA, patients were stratified into five groups: target, cytostatic, target and immunotherapy, immunotherapy, and naïve.

We retrospectively investigated, from 2012 to 2018, 578 stage IV cutaneous (excluded mucosal and ocular) melanoma patients treated with anti-CTLA-4 (ipilimumab) or anti-PD-1 (pembrolizumab or nivolumab) as monotherapy at the INT-NA (Figure 1) (Table 1) [20]. Ipilimumab was administered intravenously at a dosage of 3 mg/kg every 3 weeks for 4 doses, pembrolizumab at a dosage of 200 mg every 3 weeks, and nivolumab at a dosage of 3 mg/kg every 2 weeks, until disease progression or unacceptable toxicity appeared. Disease evaluation was performed at baseline and, subsequently, every 12 weeks until progression or the discontinuation of treatment according to the Response Evaluation Criteria in Solid Tumors (RECIST 1.1) [21]. Based on the availability of the data reported in clinical records, clinical variables such as sex, age, BRAF status, LDH, NLR, CNS (central nervous system) metastases, and eosinophils used in routine for metastatic stage IV melanoma patients are presented in Table 1. LDH values were grouped according to the local laboratory reference (LLR) interval: normal = 1× LLR; high > 1× < 2× LLR; very high > 2× LLR. The NLR was calculated by dividing the absolute counts of neutrophils by the absolute counts of lymphocytes. The range was considered normal with a ratio between 1 and 4, low < 1, and high > 4.

Anti-CTLA-4 (ipilimumab 51%) and anti-PD-1 (nivolumab 26%; pembrolizumab 23%) were defined as intervention variables for the ICI program. The distribution between females and males was equally balanced. Furthermore, patients were stratified into five groups based on the type of treatment they had received before they were included in the ICI program at the INT-NA (Figure 1, Table 1). A total of 34% percent of patients did not receive any therapy before inclusion (naïve), 25% received prior ICI treatments, 18% received prior target therapy (TT), 10% of patients received an ICI and TT, and 13% of patients were treated with cytostatic schedules. For further predictive and prognostic analyses, the types of pretreatment agents were dichotomized in no target and target subgroups.

### 2.2. Statistics

#### 2.2.1. Survival Analysis

The χ2 trend test was used to examine patient characteristics for discrete categorical variables or factors. Three time-related statistical events have been considered in this study with regard to overall survival, with a statistical event defined as death from any cause; survival time was calculated using date of treatment start and date last seen or date of death (end of follow-up). Cumulative survival plots and time-to-event curves were constructed using the Kaplan–Meier product limit method, with the log-rank test applied to detect differences between groups. Univariate Cox regression analyses were performed for each prognostic factor. Hazard ratios (HRs) and 95% confidence intervals (CIs) were estimated. To test the assumption of proportional hazards, an interaction term of a prognostic variable and a time-dependent covariate were added. A significant effect of that interaction term denotes the presence of a time-dependent effect and, thus, a violation of the proportional hazards assumption. Multivariate Cox regression analyses were performed including binary coding of all factors with a stepwise procedure. *p*-values < 0.05 were considered statistically significant. All analyses were performed with the programs StatView™ for Windows and SAS Institute Inc. Version 5.0.1.

#### 2.2.2. The Clinical Categorization Algorithm (CLICAL)

In the first step of the aggregation of the clinical variables, based on the risk power defined by the Mantel-Cox analysis, a simple algorithmic score was created. Depending on the dichotomous or trichotomous category and the result of risk value found in a multivariate test, a weight that defines a favorable (3 or 2; low) or unfavorable (1; high) risk, was given to each variable chosen to build the algorithm. The final value, named predictive score (CLICAL SCORE), was calculated by summing the weight of each variable and dividing it by the number of the variables selected:

(1) $$\mathrm{CLICAL}\;\mathrm{SCORE}=\sum_{i}^{n}ax_{i}+bx_{i}+\ldots nx_{i}/n$$

where *n* = the number of variables; *ax* *nx* = the specific variables; and *i* = the weight given: 1, 2, or 3.

In order to let the algorithm reach a high performance of prediction, all variables for each patient are expected to be given (no missing info). Based on this, 503 out of 578 patients were considered. The scores were grouped into predictive signatures (CLICAL SIGNATURE) from the worst benefit (Signature I) to the best benefit (Signature V). The variables and their relative weights were age (younger vs. older-1 and 2, respectively), BRAF (mutation vs. wild type-1 and 2, respectively), pretreatment with TT (yes vs. no-1 and 2, respectively), LDH (very high vs. high vs. normal-1, 2, and 3, respectively), NLR (abnormal vs. normal-1 and 2, respectively), and eosinophil percentage (abnormal vs. normal - 1 and 2, respectively). The CLICAL methodology was applied to an external cohort of 117 patients (103 out 117 were naïve at inclusion) recruited with the same inclusion criteria at the Department of Oncology, Karolinska University Hospital, Sweden [22]. The CLICAL could significantly separate signatures of prediction for different groups of patients with the same efficiency observed in the INT-NA cohort.

#### 2.2.3. Application of the Machine Learning Survival Random Forest Clinical Categorization Algorithm (SRF-CLICAL) and Definition of Prognostic Signatures

The original CLICAL was further analyzed for the development and validation of a “proof-of-concept” algorithm using artificial intelligence (AI) methods, in particular using machine learning through the implementation of the survival random forest (SRF) model [23].

#### 2.2.4. Cox Proportional Hazards Analysis

Univariate Cox proportional hazards (Cox PH) regression models were fitted for all eight variables listed in Table 1, with survival time being used as the outcome variable, using the R package survival v. 3.2-3 [24]. Effron approximation was used for handling tied death times. The *p*-values and hazard ratios of the models were inspected to compare the predictive abilities of the independent variables. Multivariate Cox PH models were then fitted using all eight variables. Forest plots were generated to visualize the results using the ggforest function (forest plot for Cox proportional hazards model) of the R package survminer v. 0.4.8 [25]. Cox PH model performance was assessed for seven clinical variables, after excluding sex as an insignificant variable, by dividing the dataset into training and validation sets (comprised of 80% and 20% of the cohort, respectively). The R package pec v. 2019.11.03 [26] was then used for making predictions for the validation set based on the Cox PH model, and for calculating prediction errors and C-indices. The riskRegression package v. 2020.02.05 [27] was used for plotting time-dependent ROC curves and calculating AUC values.

#### 2.2.5. Survival Random Forest Model

The SRF model was computed for the data using the following seven variables as features: age group (≤ 60 or > 60 years), BRAF mutation status, LDH levels, presence of CNS metastasis, previous treatment type, eosinophil levels, and NLR (see also Table 1). The R package randomForestSRC v. 2.9.3 [28] was used for computing the model using the training dataset (80% of the cohort). An optimized SRF model was generated by tuning mtry and node size parameters for 50, 100, 200, 500, and 1000 trees using the tune.rfsrc function of the randomForestSRC package, with the starting value of mtry set to 2. Out-of-bag (OOB) errors of the models were compared, and the number of trees with the smallest OOB error (ntree = 1000) was chosen as the ntree value for the optimized SRF model, with optimal mtry = 2 and nodesize = 10 values for the given number of trees used for generating the final model. The R package pec v. 2019.11.03 [26] function predictSurvProb was then used for making survival probability predictions for the 20% validation set at 12, 24, 36, and 60 months. The riskRegression package v. 2020.02.05 [27] was used for plotting time-dependent ROC curves as in the previous assessment of the Cox PH model’s performance. Similarly, an SRF model was also computed using the full dataset. The parameters of the optimized SRF model for the full dataset were ntree = 500, try = 2, and node size = 6.

#### 2.2.6. Kaplan–Meier Survival Curves 

Kaplan–Meier plots were generated using the R packages survival v. 3.2-3 [24] and survminer v. 0.4.8 [25] for patients divided into three risk groups based on the SRF- predicted survival probabilities. For that, the full dataset was used to generate an optimized SRF model and make predictions of survival probability for each patient. The full dataset was used for this analysis so that an adequate number of patients could be assigned to each group. Distribution of the predicted survival probabilities at 5 years (60 months) was examined and used to define the risk group categories of the patients: patients with survival probability <0.2 were categorized into the high-risk group, patients with survival probability ≥0.41 were categorized into the low-risk group, and patients with survival probability in between these thresholds were categorized into the medium-risk group. The patients were further stratified according to their treatment group (anti-CTLA-4 or anti-PD-1).

## 3. Results

### 3.1. Clinicopathological Features of Melanoma Patients

A total of 578 stage IV cutaneous melanoma patients (323 males, 255 females, median age 61.2) were included in the present study (Figure 1) (Table 1); 292 out of 578 patients (51%) received ipilimumab as monotherapy, 151 out of 578 patients (26%) received nivolumab as monotherapy, and 125 out of 578 patients (23%) received pembrolizumab as monotherapy. The clinical variables of the patients are presented in Table 1. The distribution is also specified for females and males. Additionally, the age had a cutoff at 65 years separating the group into younger and older, with a slightly higher representation among the patients aged 65 and over. In the male group, 58% were older patients compared to 42% of younger males, and the difference was not significant. For 548 patients (94.8%), the presence of BRAF mutation at the codon 600E was analyzed, while 30 patients were not tested for BRAF mutation. A total of 43% of patients had a detectable mutation in BRAF, with no statistical significance between sexes. CNS metastases were present in 28% of the cases included in the analysis, with an equal distribution between sexes. LDH values were grouped according to the local laboratory reference (LLR) interval: normal = 1× LLR; high > 1× < 2× LLR; very high > 2× LLR. The level of LDH was detected as very high in 14% of the cases, high in 20% of patients, and normal in 66% of the patients. Only 9% of patients had elevated eosinophils in their circulating blood. The NLR was abnormal in 45% of the patients. No difference between females and males was registered for these peripheral blood parameters. 

### 3.2. The Efficacy of ICI Depending on the Previous Treatment

**Figure 2:**
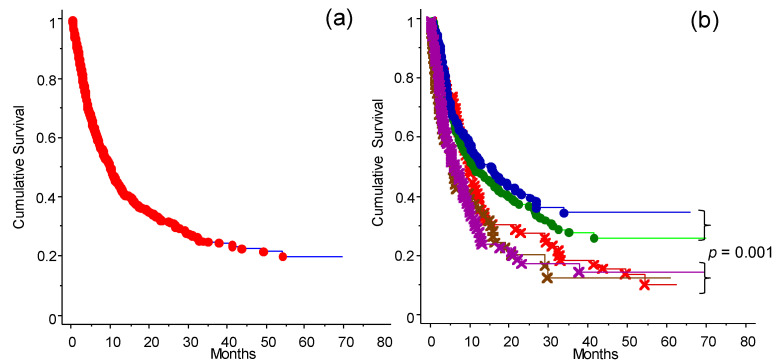
Cumulative survival analysis of patients depending on treatment before enrollment to the ICI program at the INT-NA: (**a**) Survival curve for the whole cohort of patients (*n* = 578). (**b**) Survival analysis of patients stratified into five groups based on treatment received before access to the INT-NA’s program: naïve (blue line); immunotherapy (green line), cytostatic (red line), immunotherapy and target treatment (brown line), and target treatment (purple line). Naïve and immunotherapy groups (non-target) had an OS significant higher compared to the other treatment groups (*p* = 0.001) (see also Figure 4a).

**Figure 3:**
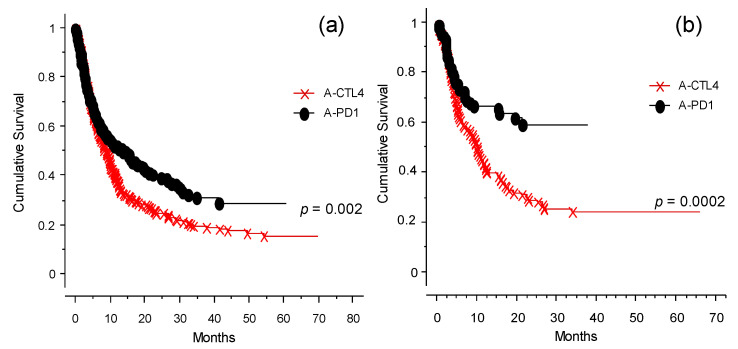
OS analysis for anti-CTLA-4- or anti-PD-1-treated patients (observation time >40 months): (**a**) OS analysis for the whole cohort of patients categorized by type of ICI received at INT-NA: anti-CTLA-4 (×--×) or anti-PD-1 (•--•), *p* = 0.002. (**b**) OS analysis of the naïve group selected for type of ICI received at INT-NA: anti-CTLA-4 (×--×) or anti-PD-1 (•--•), *p* = 0.0002.

The analysis of the OS of the population of patients studied is presented in Figure 2. Taken together (for any type of intervention ICI), the entire cohort of 578 cases had an OS of 20% at 70 months (Figure 2a). Applying the different categories of treatment (as defined in Table 1), naïve and immunotherapy pretreated patients had the highest OS (Figure 2b). The patient groups that received TT only or ICI and TT before the start of the study had the worst outcomes. OS analysis of patients included in the program at the INT-NA, categorized based on ICI treatment (anti-CTLA-4 and anti-PD-1), is shown in Figure 3. As expected, the anti PD-1 strategy showed a better impact in the final outcome compared to anti-CTLA-4 treatment (Figure 3a, *p* = 0.002), and it was particularly efficacious in naïve patients (Figure 3b, *p* = 0.0002).

### 3.3. The Response to Immunotherapy

The analysis of response to the ICIs is presented in Table 2 and Table 3. Table 2 shows the analysis of relapse-free survival (RFS) and OS for all patients, while Table 3 shows only naïve patients.

### 3.4. Analysis of the Factors Related to the Efficacy of Immunotherapy (ICI Program)

#### Role of the Different Treatments Given before Inclusion to the INT-NA

**Figure 4:**
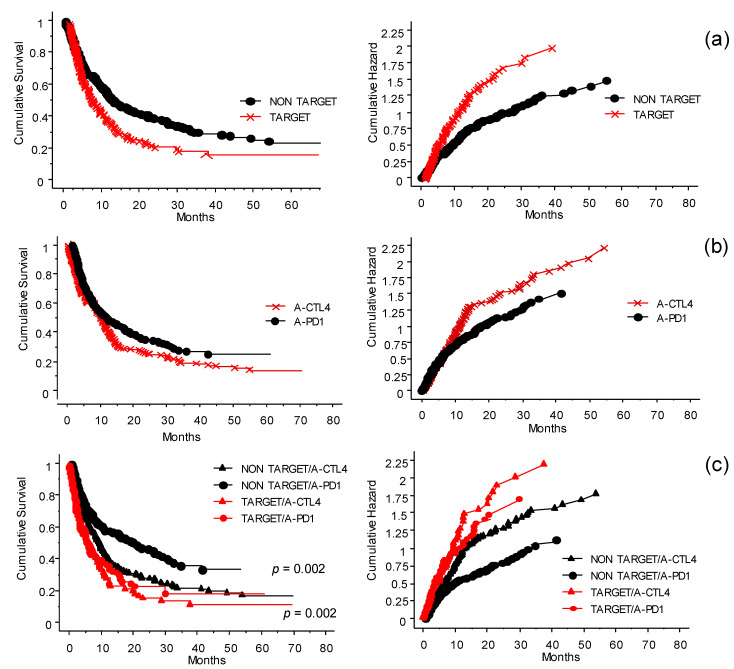
Interaction and significance of the treatments received before inclusion in the ICI program at the INT-NA: cumulative survival (left panels) and hazard plots (right panels). (**a**) The treatments before inclusion (see Figure 2b) have been simplified by grouping them into target (red line) or non-target (black line) groups. Patients who did not receive target treatment had more favorable outcomes (*p* < 0.0001). (**b**) In the cohort of patients who received prior TT, when exposed to anti-CTLA-4 (×) or anti-PD-1 (●) (i.e., naïve not included), interestingly, the effect of anti-PD-1 was not significantly different from that of anti-CTLA-4 (*p* = 0.07). (**c**) Significance of first-line treatment with target or non-target therapy on the outcome of the ICI program. In both anti-CTLA-4 (**∆**) and anti-PD1 (●) treatments, patients who did not receive previous target treatment had a more favorable outcome (*p* = 0.002).

In Figure 4 the role of the treatments received by patients before treatment with ICI at the INT-NA is shown, with particular regard to TT. The previous treatments have been grouped in target or non-target (Figure 4a). The group of patients that received TT responded poorly (*p* < 0.0001). Interestingly, in this group the effect of anti–PD-1 was not significantly different from that of anti-CTLA-4 (Figure 4b, *p* = 0.07). Looking at anti-CTLA-4- and anti-PD-1-treated patients, those who did not receive previous TT had more favorable outcomes in both cases (Figure 4c, *p* = 0.002).

### 3.5. Treatment of Patients Who Relapsed after the ICI Program at the INT-NA

**Figure 5:**
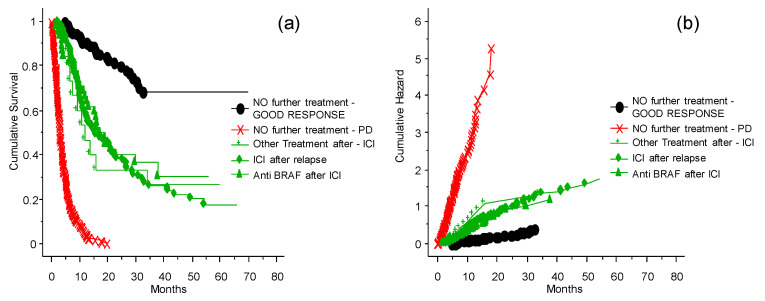
The probability of survival and risk for patients who relapsed after the ICI program at the INT-NA. Panel (**a**) shows probability of survival, panel (**b**) shows risk. Subsequent line of treatment or no further treatment, and comparison between TT given after the ICI program.

The probability of survival and chance of good response for patients without further treatment was significantly different (Figure 5, *p* = 0.0001) from other intervention strategies (treatments other than ICIs and TT after relapse, ICIs after relapse, TT after relapse, or no further treatment) (Figure 5). It is also important to note that the patients treated with other therapies after disease relapse had a clinical benefit compared to patients who were not treated after relapse. There is no evidence of differences between ICIs or other strategies of treatment after relapse. Thus, when possible, it is advantageous to invest in further treatments.

**Figure 6:**
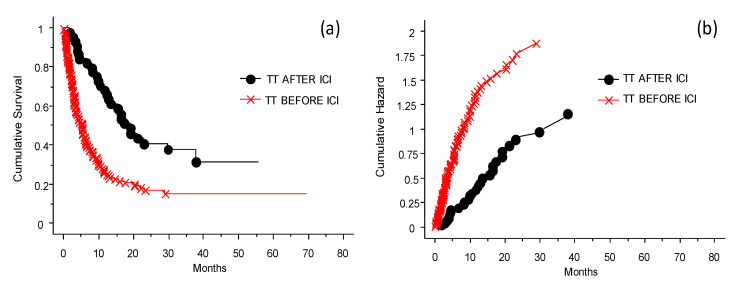
Probability of survival and risk for patients treated with TT before or after ICIs. Panel (**a**) shows probability of survival, panel (**b**) shows risk. The patients had different benefits depending on whether the treatment with TT was delivered before (worst OS, red line) or after (better OS, black line) the challenge with ICIs in case of disease progression (*p* < 0.0001).

The results summarized in Figure 6 needs to be scrutinized looking at the presence or absence of BRAF 600E and consequent treatment or not before exposure to ICIs. In fact, patients who were treated with TT prior to ICIs had less opportunity to respond to ICIs, whether it was anti-CTLA-4 or anti-PD-1 therapy (see also Figure 4). The effect of anti-PD-1 was not significantly different from that of anti-CTLA-4. Treatment with anti-PD-1 of patients previously treated with cytostatic drugs or immunotherapy, or naïve patients, produced a better OS. The patients had different benefits depending on whether the treatment with TT was delivered before or after challenge with ICIs (*p* < 0.0001).

### 3.6. Analysis of the Predictive Power of Clinical Variables at Inclusion 

**Figure 7:**
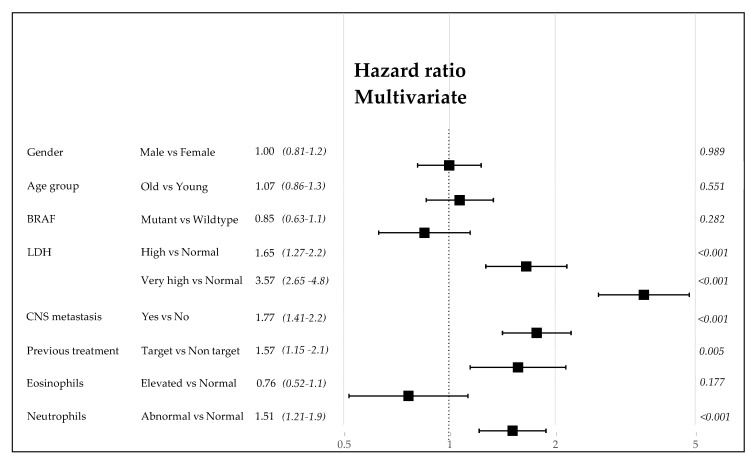
Forest plot of the clinical variables. The hazard ratio and 95% CI are described as well as the *p*-value.

**Figure 8:**
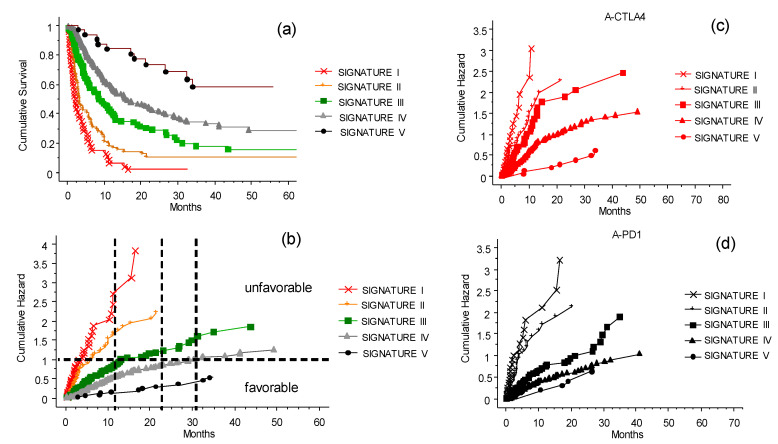
The CLICAL signatures and their interaction with the ICI treatments. Plot (**a**) shows the cumulative survival with the distribution of the patients grouped by their signatures. The five curves are significantly separated from one another (*p* = 0.001). (**b**) Cumulative risks for each signature easily show whether the characteristics of each signature are favorable or not. Plot (**c**) presents the effect of the signature when compared with anti-CTLA-4 treatment. The difference in prediction for the five signatures is significant (*p* = 0.0001). (**d**) Signatures IV and V predict a similar benefit to patients treated with anti-PD-1, and are different from Signature III as well as from I and II (*p* = 0.0001).

The relevant clinical data given by gender, age, BRAF 600E mutation, LDH, CNS metastases, previous TT before the inclusion to ICI program, eosinophil counts, and NLR were initially analyzed for their risk of death due to the metastatic disease via univariate and multivariate Mantel-Cox methods (as explained in the Materials and Methods section). Each of these variables could determine, singularly or in a hierarchical way, the power of prognosis as shown in the forest plot (Figure 7). This preliminary analysis permits us to assign the weight of risk and build the score derived from the CLICAL verified by the SRF-CLICAL algorithm (Table 4). Based on hazard ratio, gender was subsequently excluded from the CLICAL. The algorithm calculated eight score levels; these were scrutinized in a survival plot, and those closer to one another were grouped together for the final five signatures (Table 4 and Figure 8a).

### 3.7. The CLICAL Signature and Prediction of Survival Rates

The CLICAL algorithm has the ability to distinguish groups of patients by their signature. The five signatures had different prediction rates for survival (Figure 8a,b) (*p* = 0.001); the higher the signature, the better the odds of survival. Signature I, which is built by the highest risk values for each of the variables selected in the algorithm, had no survival after 32 months. On the opposite end, the patients with Signature V, built by the lowest risk values for each variable, had the highest percentage of survival. The plots separated by the different signatures shown in Figure 8a,b represent the separation of the survival curve of the whole cohort after the ICI program at the INT-NA, as presented in Figure 2a. The difference in prediction between the signatures is significant (*p* = 0.0001). Looking at anti-CTLA-4 therapy (Figure 8c), the signatures show clearly that patients with lower signatures (I–II) will not benefit from the treatment compared to patients with Signature V. Still, there is a possibility of a longer survival in the group with Signature IV. For comparison, see the prognostic plot for the whole cohort presented above in Figure 3.

The same could be said for the anti-PD1 therapy (Figure 8d), with the difference being that Signature III–V indicates a significant benefit from the therapy. Signatures IV and V give similar prediction, and significant difference to that of Signature III. Signatures I and II were not associated with a long-lasting benefit. In practice, the use of the signature can provide a tool to decide whether it is beneficial to expose a patient with the lowest signature to both of the intervention ICIs as a second challenge, or instead concentrate on more palliative strategies, avoiding the ICI side effects.

### 3.8. The CLICAL Signature and Prediction of Response to ICIs

**Figure 9:**
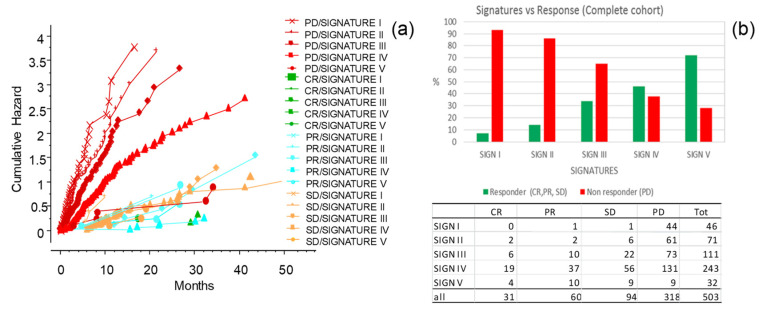
The CLICAL signatures and response to the ICI treatment (complete response (CR), partial response (PR), stable disease (SD); responders), (progressive disease (PD); non-responders). (**a**) Cross-analysis of the CLICAL signatures panel with the results from ICI intervention. Cumulative risks for each signature easily show whether the characteristics of each signature are favorable or not. (**b**) Percentage of cases in responders present in each signature after the ICI treatment. Signature V comprises a higher percentage of responder patients and a lower percentage of non-responder patients compared to Signature I. The numbers of cases for each response category and signature are presented in the attached table.

The CLICAL algorithm has the ability to distinguish response to ICI treatment of patients by their signature. The five signatures had different prediction rates for response (Figure 9a,b) (*p* = 0.001). Signature I had lower rates of response and, at the opposite end, the patients with Signature V had the highest percentage of response; the higher the signature, the better the odds of response. The cumulative hazard plot shows that patients not responsive to the therapy also have a higher risk to die earlier compared to patients responding to the therapy (Figure 9a). Interestingly, non-responding patients but with higher signatures (Signatures IV and V) have a better chance to survive compared to non-responding patients with lower signatures (Signatures I, II, and III) (Figure 9a). The group of patients with higher signatures (i.e., less and less risk), is composed of an increasing percentage of responsive patients and a decreasing percentage of non-responsive patients compared to the group of patients with lower signatures (Figure 9b). Of interest is the fact that patients who did not respond to the ICI treatment could still have a chance to live longer if they had a higher signature at inclusion; this could be due to the opportunity to receive subsequent treatment.

### 3.9. The CLICAL Signature Applied to an External Cohort

**Figure 10:**
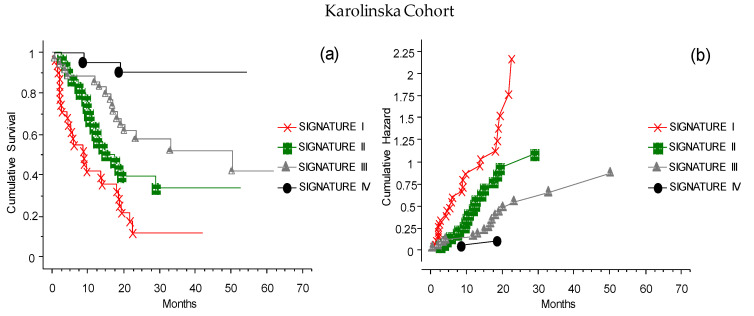
The CLICAL signatures obtained from the external cohort from Karolinska. Due to the restricted numbers, the cases could only be grouped in Signatures I–IV. (**a**) The plot shows the cumulative survival, with the distribution of the patients grouped by their signatures. The four curves are significantly separated from one another (*p* = 0.001). (**b**) The presentation of the cumulative risks for each signature easily shows that the CLICAL can determine the characteristics of each signature.

The analysis with CLICAL was also applied to an external cohort of 117 patients, available at the Karolinska University Hospital, Sweden. The same categorical variables were studied, and the signatures obtained significantly discriminated the predictive benefits of ICI treatment (Figure 10). In this cohort, the CLICAL could define only four signatures (Signatures I–IV), since the number of available patients with the highest score (only one patient) was not sufficient to build five levels of signature.

### 3.10. The Validation of the CLICAL Algorithm’s Efficiency by Machine Learning Survival Random Forest Analysis (SRF-CLICAL)

**Figure 11:**
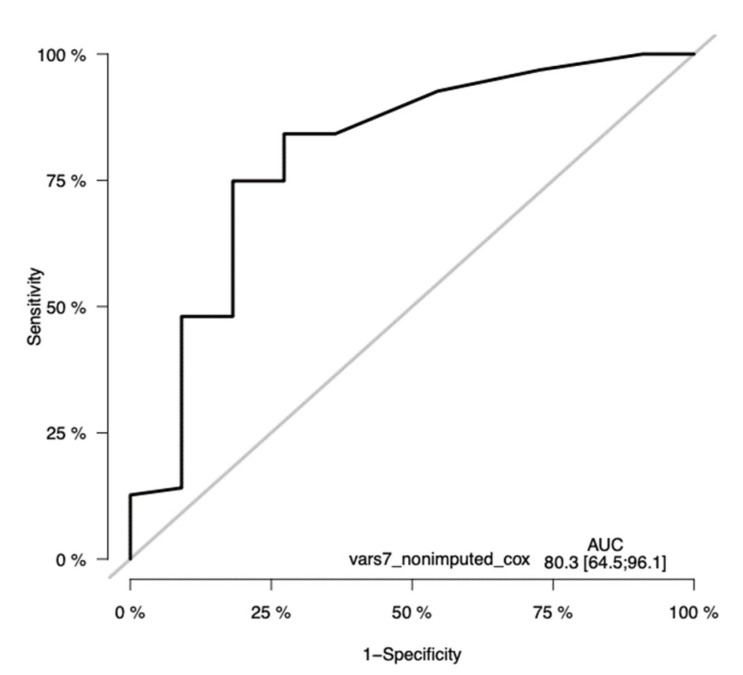
Time-dependent receiver operating characteristic (ROC) curves of the seven-variable Cox PH model at the timepoint of 36 months. The ROC has a significant sensitivity, with an AUC of 80.23(CI 64.5–96.1). The y-axis in the plot displays the true positive rate (TPR)—i.e., sensitivity—whereas the x-axis in the plot displays the false positive rate (FPR), i.e., specificity.

Prediction performance of the Cox model with seven variables (gender was excluded from the model) was studied using training–validation settings, and time-dependent receiver operating characteristic (ROC) curves at time points 12, 24, 36, and 60 months were generated (Figure 11). The resulting areas under the ROC curves (AUCs) were computed, and are also shown in the plots. As shown in the plots (Figure 11), the AUCs of the seven-variable Cox models were 71.5, 73.5, and 80.3 at timepoints 1, 2, and 3 years, respectively. At the timepoint of 5 years, the number of cases was so low that a proper, informative ROC curve could not be computed.

### 3.11. Survival Random Forest Model

**Figure 12:**
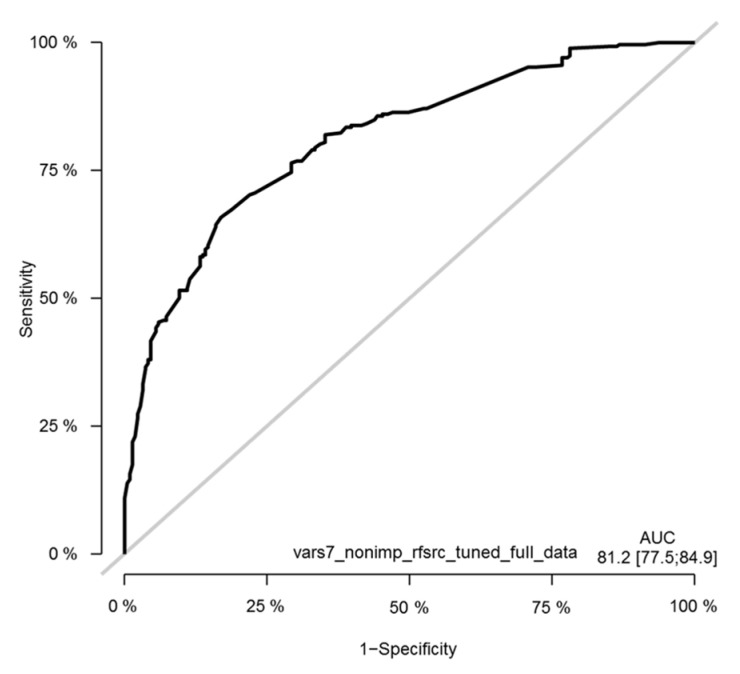
Time-dependent receiver operating characteristic (ROC) curves of the tuned seven-variable SRF model of the melanoma cohort. The ROC curve is shown at the timepoint of 36 months. The y-axis in the plot displays the true positive rate (TPR)—i.e., sensitivity—whereas the x-axis in the plot displays the false positive rate (FPR), i.e., specificity.

Survival random forest (SRF) models were created for predicting patient survival using the same seven clinical variables as the selected features for the models that were used for the Cox model. Optimized SRF models were generated by tuning model parameters and by using similar training—validation settings as for the Cox model, as well as using the full dataset. Due to the available sample size, the ROC curves were generated using the full dataset to obtain an adequate number of patients (Figure 12).

### 3.12. The SRF-CLICAL Signature

**Figure 13:**
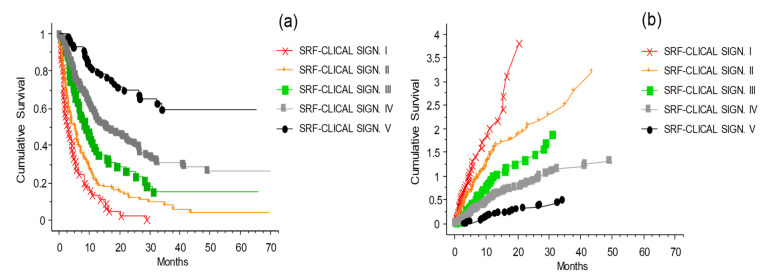
Kaplan–Meier survival curves of the melanoma patients divided into five risk groups based on their survival probability predictions obtained using the optimized survival random forest model. Plot (**a**) shows the cumulative survival with the distribution of the patients grouped by their signatures (applied to the risk groups defined by the SRF-CLICAL). The five curves are significantly separated from one another (*p* < 0.0001). (**b**) The presentation of the cumulative risks for each signature easily shows whether the characteristics of each signature are favorable or not.

To validate the usage of the SRF model for predicting melanoma patient outcomes, the patients were divided into five risk groups—very high risk (Signature I), high risk (Signature II), medium risk (Signature III), low risk (Signature IV), and very low risk (Signature V)—based on their SRF-predicted survival probabilities. Survival curves for these five signatures were then compared (Figure 13). The five risk groups showed clearly and statistically significantly distinct survival curve profiles (*p* < 0.0001). These results are consistent, and validate the original simplified CLICAL signatures definition as shown in Figure 8.

## 4. Discussion

Cutaneous melanoma is the most aggressive form of skin tumor, and its incidence has significantly increased in recent decades [29]. Fortunately, over the past few years, the development of immunotherapy with ICIs and TT against kinases of the RAS/BRAF/MAPK pathway has dramatically improved its clinical outcomes, with the achievement of long-term benefit in approximately 50% of patients with metastatic disease, completely changing the perspective for melanoma patients [30,31,32]. Immunotherapy has played a primary role due to the availability of new monoclonal antibodies directed toward the checkpoint molecules CTLA-4 and PD-1 [7]. The anti-CTLA-4 antibody (ipilimumab) can induce a response rate of approximately 15%, with approximately 20% of patients being long-term responders [32]. The anti-PD-1 drugs (nivolumab or pembrolizumab) have shown a higher response rate of approximately 40% in treatment-naïve patients, with the majority of responses being durable [33]. However, the main limitations affecting the use of these agents are represented by the heterogeneous response of patients, and by the absence of universally recognized predictive biomarkers of response [34,35]. Based on these observations, in this study we have analyzed real-world data related to cutaneous metastatic melanoma patients treated with ICIs. To provide a useful tool for helping clinicians to make the best therapeutic decisions, we built an algorithm including patients’ baseline clinical characteristics, investigating whether it could predict the response to treatment. 

We retrospectively investigated 578 metastatic melanoma patients who received ipilimumab (51%), pembrolizumab (23%), or nivolumab (26%) as monotherapies at the INT-NA. The whole cohort of 578 cases analyzed had an OS of 23% at 70 months (median 10 months; CI 95%: 8.4–11.2); meanwhile, analyzing patients grouped by treatment received at the INT-NA, ipilimumab-treated patients had an OS of 15% at 60 months (median 8.9 months; CI 95%: 7.1–10.2), nivolumab-treated patients had an OS of 29.4% at 60 months (median 15.7 months; CI 95%: 9.5–26.5), and pembrolizumab-treated patients had an OS of 25% at 60 months (median 11 months; CI 95%: 7.2–16.2). Our results are consistent with clinical efficacy evidence derived from other real-life studies [6,22,31,32,34]. Moreover, we defined five groups of patients based on the therapy received before treatment at the INT-NA: 24% were naïve, 25% received immunotherapy, 18% received TT, 13% were treated with cytostatic agents, and 10% received both TT and immunotherapy. Among these five groups, naïve and immune-pretreated patients had the highest survival, while patients pretreated with TT had the worst outcomes. Furthermore, data from other real-life retrospective analyses confirm our observations [22,36,37]. We proceeded to refine the analysis by grouping patients according to whether they had previously received TT or not. The group of patients who received TT responded poorly to the ICI treatments, and the effect of anti-PD-1 was not significantly different from that of anti-CTLA-4. In fact, patients who did not receive previous target treatment had more favorable outcomes in both cases. In addition, we analyzed the effect of target treatment on immunotherapy based on whether it was delivered before or after ICI treatment. The patients treated with TT after challenge with ICIs had a better OS compared to patients treated with ICIs after TT. Moreover, increasing evidence from the literature seems to be in accordance with our results. However, the most effective sequence of these agents has not been well characterized, although several studies were conducted to help make the best decisions for our patients [37,38,39].

Afterwards, the clinical variables routinely used to define the general status of disease for metastatic melanoma were analyzed for their risk of death or relapse [16,22,40,41,42]. Once we analyzed all of the clinical parameters, we could define and validate the prediction algorithm CLICAL which, based on the different score and relative signature attributed to each patient, is able to determine the degree of benefit obtained with ICI treatment for metastatic melanoma patients (Figure 8 and Table 4). This study shows that there is a group of patients where Signature V predicts an excellent response to the treatment, wherein more than 50% of the patients would still be alive at 70 months, while at the opposite end there is a group of patients where Signature I predicts no response. It should be noted that CLICAL Signatures IV and V include patients with high probability of survival regardless of the type of ICI. This means that patients treated with anti-CTLA-4 or anti-PD-1 who have the highest scores have the same opportunity of response. On the other hand, the lowest signatures (I and II), regardless of the ICI, predict a very low or no benefit at all. This discriminant model raises the question of whether having this knowledge justifies commencing a treatment with the intention of obtaining lasting clinical benefit. To validate the potential prognostic role of the CLICAL, the algorithm was applied to an external cohort of 117 patients recruited at Karolinska University Hospital, Sweden. In this case, the CLICAL also identified different groups of patients, depending on outcome, with the same efficiency as observed in the INT-NA cohort. The use of signatures to determine categorical variables is increasing [20,43,44,45]. The clear distinction of the input and quality of information is relevant (patient’s clinical variable, intervention, and time variable). Lastly, we introduced the machine learning SRF method to ensure that the CLICAL could identify a signature with high predictive power. The SRF-derived signature correlates to and visualizes the group selected by the original CLICAL empirical algorithm [23]. This knowledge might change future approaches to determining who to treat. In clinical praxis, this could also be applied in prospective clinical studies considering known validated variables for potential inclusion in the algorithm, and could predict the outcome of the individual patient depending on the defined signature at the beginning of ICI treatment.

## 5. Conclusions

In conclusion, with all of the limitations of a retrospective population-based study, we provide evidence that the analysis of real-life treatment of metastatic malignant melanoma patients reveals the possibility to increase the OS with ICI products. The collection of clinical parameters is an important tool in the analysis of their predictive power. In fact, this study shows that the application of the CLICAL and SRF-CLICAL algorithms can characterize individual patients with different benefits from ICI treatment. These prediction algorithms are likely to be useful for decision-making on ICI referrals, and to facilitate decisions on the eligibility of each patient with metastatic melanoma to enter the ICI program at the INT-NA. From the results of this study, we can ensure that the right patient receives the right treatment, which will benefit both the individual patient as well as the decision-making doctor.

## Figures and Tables

**Table 1:** Clinical–pathological characteristics of cutaneous melanoma patients. Description of the patient population and inclusion criteria adapted to the real-life situation.

Variables	All		Females		Males	
	*n*	%	*n*	%	*n*	%
Clinical anamnestic variable						
Sex	578	100	258	45	320	55
Median age (range)	61.4 (23–89)		59.9 (23–89)		62.5 (22–87)	
Age 65–100	317	55	132	51	185	58
Age 18–64	261	45	126	49	135	42
BRAF mut 600E tested	548 ^a^	100	241	100	307	100
BRAF 600E mutated	234	43	108	45	126	41
CNS met	572	100	257	100	315	100
Yes	162	28	71	28	91	29
No	410	72	186	72	224	71
LDH	535	100	241	100	294	100
Very high (>2 × LLR)	77	14	39	16	38	13
High (≥1 × ≤2 × LLR)	107	20	43	18	64	22
Normal (<1× LLR)	351	66	159	66	192	65
Eosinophil counts	543	100	242	100	301	100
Elevated	47	9	18	7	29	10
Normal	496	91	224	93	272	90
NLR	551	100	248	100	303	100
Abnormal	247	45	104	42	143	47
Normal	304	55	144	58	160	53
**Treatment groups**						
None (Naïve)	199	34	83	32	116	36
Immunotherapy	142	25	61	24	81	25
Target and immunotherapy	59	10	29	11	30	9
Target	102	18	48	19	54	18
Cytostatic	76	13	37	14	39	12
**Grouped in Pre-target /No target**						
No target	417	72	181	70	236	74
Pre-target	161	28	77	30	84	26
**Treatment at inclusion to the INT-NA ICI program**						
Anti-CTLA-4	292	51	129	50	163	51
Anti-PD-1 (nivolumab)	151	26	68	26	83	26
Anti-PD-1 (pembrolizumab)	135	23	61	24	74	23

^a^ 30 patients lack test results for BRAF 600E mutation: naïve (9), cytostatic (22), or immunotherapy (5); hence, none of them received target therapy before inclusion.

**Table 2:** RFS and OS analysis of the whole cohort. Probability of relapse free survival and probability of survival after immunotherapy received at inclusion at the INT-NA.

		RFS				OS			
	*n* (%)	Probability of RFS (%) at 24 Months	CI 95% Min–Max	Cumulative Hazard at 24 Months	CI 95% Min–Max	Probability of Survival (%) at 50 Months	CI 95% Min–Max	Cumulative Hazard at 50 Months	CI 95% Min–Max
ALL	578 (100)	16	13–19	1.83	1.63–2.03	8	5–11	2.46	2.12.80
CR	32 (5.5)	67	48–85	0.39	0.12–0.66	81	61–100	0.19	0–0.43
PR	74 (12.8)	64	52–76	0.48	0.28–0.67	58	42–75	0.52	0.24–0.79
SD	103 (17.8)	24	15–33	1.48	1.09–1.86	39	25–52	0.94	0.60–1.28
PD	369 (63.8)	0	0	6.42	3.91–8.93	5	2–7	2.9	2.4–3.4

**Table 3:** RFS and OS analysis of naïve cohort. Probability of relapse free survival and probability of survival after immunotherapy received at inclusion at the INT-NA.

		RFS				OS			
	*n* (%)	Probability of RFS (%) at 24 Months	CI 95% Min–Max	Cumulative Hazard at 20 Months	CI 95% Min–Max	Probability of Survival (%) at 50 Months	CI 95% Min–Max	Cumulative Hazard at 50 Months	CI 95% Min–Max
ALL	199 (100)	22	16–28	1.49	1.22–1.76	36	27–42	1.06	0.83–1.28
CR	17 (8.5)	81	62–100	0.32	0–0.66	100	100	0	0
PR	29 (14.5)	73	56–90	0.31	0.08–0.53	76	52–99	0.27	0–0.56
SD	36 (17.6)	31	14–43	1.13	0.61–1.64	55	34–76	0.58	0.21–0.96
PD	118 (59.3)	0	0	4.28	2.73–5.83	11	6–17	2.16	1.61–2.71

**Table 4:** CLICAL algorithm scores and signatures related to 503 out of 578 patients. CLICAL algorithm outcomes in scores and grouped in signatures defined by the cumulative risk at 32 months.

CLICAL SCORE	*n*	Cumulative Hazard at 32 Months (95% CI)	Still Alive (at Risk) at 32 Months	CLICAL SIGNATURES	*n* (%)	Cumulative Hazard at 32 Months (95% CI)
1.143	8	2.7 (0.29–5.14) ^#^	0	Signature I	46 (9.1)	3.42 (1.87–4.96)
1.286	38	3.23 (1.69–4.77)	1			
1.429	71	2.41 (1.59–3.24)	2	Signature II	71 (14.1)	2.41 (1.59–3.24)
1.571	111	1.70 (1.24–2.16)	10	Signature III	111 (22.1)	1.70 (1.24–2.16)
1.714	127	1.14 (0.86–1.43)	10	Signature IV	243 (48.3)	1.08 (0.89–1.27)
1.857	116	1.00 (0.75–1.20)	30			
2.000	31	0.50 (0.19–0.81)	12	Signature V	32 (6.4)	0.48 (0.19–0.77)
2.143	1	0.50(0.19–0.81)	1			

^#^ 100 % dead at 16 months.

## Data Availability

The datasets used and/or analyzed during the current study are available from the corresponding author on reasonable request.
